# Comparison of three different prophylactic treatments for postoperative nausea and vomiting after total joint arthroplasty under general anesthesia: a randomized clinical trial

**DOI:** 10.1186/s40360-024-00735-9

**Published:** 2024-01-30

**Authors:** Jinwei Xie, Yingcun Cai, Fuxing Pei

**Affiliations:** 1 Department of Orthopaedic Surgery, West China Hospital, Sichuan University, 37#Guoxue Road, Chengdu, Sichuan Province 610041 People’s Republic of China; 2 Department of Orthopaedic surgery, The First Affiliated Hospital of Zhengzhou University, No.1 East of Jianshe Road, Zhengzhou, 450052 People’s Republic of China

**Keywords:** Postoperative nausea and vomiting, Arthroplasty, Enhanced recovery after surgery

## Abstract

**Background:**

Postoperative nausea and vomiting (PONV) after total joint arthroplasty is common and associated with delayed recovery. This study was performed to evaluate the efficacy of three different prophylactic regimens for PONV after total joint arthroplasty under general anesthesia.

**Methods:**

Patients undergoing primary total hip or knee arthroplasty were randomized to Group A (ondansetron), Group B (10 mg dexamethasone plus ondansetron and mosapride), or Group C (three doses of 10 mg dexamethasone plus ondansetron and mosapride). The primary outcome was the total incidence of PONV during postoperative 48 h. The secondary outcomes were complete response, rescue antiemetic treatment, opioid consumption, time until first defecation, postoperative appetite score, satisfaction score, length of hospital stay, blood glucose level, and complications.

**Results:**

Patients in Group C experienced a lower incidence of total PONV (29.3%, *p* = 0.001) and a higher incidence of complete response (70.7%, *p* = 0.001) than did patients in Group A (51.9%, 48.2%, respectively). Patients in Group C also experienced a lower incidence of severe PONV (4.3%) than patients in Group A (25.9%, *p*<0.001) and B (20.4%, *p*<0.001). Moreover, less rescue antiemetic treatment (1.4 ± 0.5 mg Metoclopramide) and postoperative opioid consumption (1.8 ± 0.3 mg Oxycodone, 6.0 ± 1.0 mg Pethidine) was needed in Group C. Additionally, a shorter time until first defecation, shorter length of stay, and better postoperative appetite scores and satisfaction scores were detected in patients in Group C. A slight increase in the fasting blood glucose level was observed in Group C, and the complications were comparable among the groups.

**Conclusion:**

Combined use of ondansetron, mosapride and three doses of dexamethasone can provide better antiemetic effectiveness, postoperative appetite, bowel function recovery, and pain relief than a single dose or ondansetron only.

**Trial registration information:**

The protocol was registered at the Chinese Clinical Trial Registry (ChiCTR1800015896, April 27, 2018).

**Supplementary Information:**

The online version contains supplementary material available at 10.1186/s40360-024-00735-9.

## Background

Postoperative nausea and vomiting (PONV) are one of the most common and distressing complications after surgery. This is especially true in patients undergoing general anesthesia, among whom the incidence of PONV may reach 25- 30% and even rise to 80% among patients without prophylactic intervention [1, 2]. Optimal management of PONV is important for rapid recovery after joint arthroplasty because effective treatments that limit post-operative nausea allow patients to mobilize earlier, take food earlier, and improve overall patient satisfaction [3].

Numerous precautionary measures have been taken in an attempt to prevent PONV, such as administration of 5HT_3_ receptor antagonists, neurokinin 1 receptor antagonists, corticosteroids, butyrophenones, and antihistamines [4]. Although the efficacy of these prophylactic drugs has been confirmed by high-level evidence, PONV remains a persistent problem [5]. One reason for this phenomenon is the huge gap between implementation and the “PONV-free” goal. Another reason is the side effects associated with anti-PONV treatments, and no ideal regimen has been established. For example, use of the 5-HT_3_ receptor antagonist ondansetron can aggravate the postoperative constipation which occurs in up to 57% of patients who undergo total joint arthroplasty [6]. Dexamethasone, another common antiemetic agent, has effects on reducing the incidence of PONV in total joint surgery [7–9]. Meantime, concerns about infection safety has aroused because of its suppressive effect on immune system and increase in blood glucose level, although the current evidence doesn’t support this [10]. Furthermore, the most appropriate dosage and timing of dexamethasone have not been determined. A single dose was routine practice against PONV, and extended prevention may provide additional analgesic effect.

The previous study showed that intravenous use of opioids was risk factors for PONV following primary total hip or knee arthroplasty (odds ratio = 2.052, *p* = 0.008; published in Chinese) [11] because these drugs inhibit gastrointestinal peristalsis. In contrast, selective 5-HT_4_ agonists can stimulate the gastrointestinal tract and promote motility [12], and mosapride, one type of 5-HT_4_ agonist, is reportedly effective in reducing the incidence of vomiting caused by chemotherapy [13]. To the best of our knowledge, however, very few studies have evaluated and compared the antiemetic efficacy of mosapride with that of other types of PONV prophylaxis.

Thus, in the present study, we compared the anti-emetic efficacy of concurrent use of different doses of dexamethasone and mosapride with ondansetron. We hypothesized that combined application of three doses of dexamethasone and mosapride would provide a greater clinical benefit.

## Patients and methods

### Study design

Patients receiving primary total hip or knee arthroplasty from November 2017 to December 2018 were included. Institutional review board approval (2012 − 268) was obtained before patient enrollment. Written informed consent and research authorization were obtained from all patients before surgery. The study was conducted in compliance with the tenets of the Declaration of Helsinki, and reported in accordance with CONSORT statement. The original protocol was registered at the Chinese Clinical Trial Registry (ChiCTR1800015896, April 27, 2018).

The study was a little different from the registered protocol, regarding the exclusion criteria and samples size. Firstly, a 50% reduction in PONV was expected, which was the calculation basis of sample size in registered protocol. However, during the pilot study, patients in group B and C also experienced a high incidence of PONV, then we adjusted the expected reduction to 45% with the approval of monitoring committee.

### Inclusion and exclusion criteria

Eligible patients included those aged from 40 to 80 years old who were at risk of PONV (at least 1 score of Apfel), and scheduled for primary total hip or knee arthroplasty because of end-stage joint diseases. Exclusion criteria were as follows: having a history of intolerance to any drug used in this study, taking another antiemetic drug or systemic steroid within 24 h before surgery, having a history of allergy to the experimental drug or adverse reactions, poor control of blood sugar in diabetes, having a history of steroid or immunosuppressive drug use within the first 6 months, and a history of heart disease, such as heart failure, heart conduction block, ventricular arrhythmia or severe impairment of intestinal motility, renal function or liver function.

### Randomization and treatment

All included patients in this triple-blinded study were randomly allocated to three groups of prophylactic treatment for PONV using a computer-generated randomization list in a 1:1:1 ratio. The group assignments were performed by a research assistant and kept in opaque sealed envelopes that were only opened immediately before surgery.

- Group A received three doses of 2 ml normal saline at anesthetic induction and 6 and 24 h later; and oral placebo 3 h before surgery and three times per day after the operation.
- Group B received one dose of 10 mg dexamethasone (2 ml) at anesthetic induction, two doses of normal saline 6 and 24 h later, 5 mg oral mosapride 3 h before surgery and three times per day after the operation.
- Group C received three doses of 10 mg dexamethasone (2 ml) at anesthetic induction and 6 and 24 h later, 5 mg oral mosapride 3 h before surgery and three times per day after the operation.

All patients received 8 mg ondansetron (4 ml) at the end of surgery for primary prevention of PONV. The intraoperative dexamethasone and ondansetron were administered by an anesthesiologist, and the postoperative oral drugs were administered by nurses who were not involved in the study. The patients, surgeons, data collectors, and analysts were all blinded to the group assignments.

### Anesthesia and perioperative management

All surgeries were performed by the same team (F.X.P.) under general anesthesia, using a standard medial patellar joint incision for total knee arthroplasty and a posterior lateral approach for total hip arthroplasty. Without the use of a tourniquet, all patients undergoing total knee arthroplasty were implanted with cement stabilized prostheses, and all patients undergoing total hip arthroplasty used non cement acetabular and femoral components.

All patients were managed according to the enhanced recovery after surgery (ERAS) program. All patients received preoperative oral hydration for up to 2 h prior to surgery and adequate intravenous fluids intraoperatively. The general anesthetic regimen and multimodal pain management protocol consist of periarticular infiltration analgesia with ropivacaine, oral or intravenous nonsteroidal anti-inflammatory drugs were consistent in all participants, which have been described in our previous study in detail [14]. Once the Numeric Rating Scale (NRS) pain score was of > 4 points, 10 mg oral oxycodone was administered; if was of > 6 points, 50 mg of pethidine was given by intramuscular injection as required up to every 6 h.

#### Primary and secondary outcomes

The primary outcome was the incidence of total PONV. The incidence of total PONV was determined during the 48-hour study period by calculating the proportion of patients who experienced PONV at least once. Furthermore, newly developed PONV in each of the four periods (0 ~ 6, 6 ~ 12, 12 ~ 24, and 24 ~ 48 h) was also calculated. Following institutional guidelines, 10 mg of intramuscular metoclopramide was used as a first-line antiemetic rescue treatment when patients experienced two or more episodes of PONV within 2 h. This was followed by administration of 4 mg ondansetron intravenously when two consecutive boluses of metoclopramide alone administered at a 30-minute interval were ineffective. The episodes of nausea, vomiting and complete response were recorded and classified according the definition scoring algorithm of PONV [14, 15], by the blinded investigator (Y.C.C).

The secondary outcomes were complete response, times until first defecation and ambulation, postoperative appetite score on postoperative days 0 to 2, satisfaction score, length of hospital stay, blood glucose level, and complications. *The patients’ appetite was scored by comparison with the preoperative level (the morning on the day 1 before surgery); 1 point represented a worse appetite, 2 points represented no change in appetite from the preoperative state, and 3 points represented a better appetite.* Patients’ satisfaction before discharge was estimated using a NRS that ranged from 0 points (extremely dissatisfied) to 10 points (very satisfied). The fasting blood glucose level was measured in all patients on postoperative days 1 and 2, while the 2-hour postprandial blood glucose level was measured in patients with diabetes mellitus on postoperative day 1. All patients were followed up for 3 months postoperatively, and any complications such as prolonged QT syndrome, wound discharge, surgical site infection, or readmission were recorded.

### Statistical analysis

We performed an *a priori* power analysis based on our preliminary results showing that the incidence of PONV was 49% in patients receiving ondansetron prophylaxis alone after total joint arthroplasty [11]. We calculated that 303 patients (101 in each arm) were required to detect a 45% reduction in the incidence of PONV in Group C at an alpha level of 0.05 and power of 0.9 using a two-sided test. To allow for exclusions and dropouts, we enrolled 348 patients in the current trial.

The chi-square test or Fisher’s exact test was used to determine the statistical significance of differences in the categorical variables, such as the incidence of PONV and proportion of patients with complete response; if significant, multiple comparisons between groups were performed by Bonferroni’s corrected *post hoc* test (Z test). Continuous variables were analyzed with one-way analysis of variance if normality test was affirmative (body mass index, length of hospital stay, and times until first defecation or ambulation) or the Wilcoxon signed-rank test (patients’ appetite and satisfaction scores); if significant, multiple comparisons between groups were performed by the *post hoc* Tukey test. A p value of < 0.05 was considered statistically significant. Statistical analysis was conducted using SPSS 21.0 (IBM Corp., Armonk, NY, USA).

## Results

**Fig. 1:**
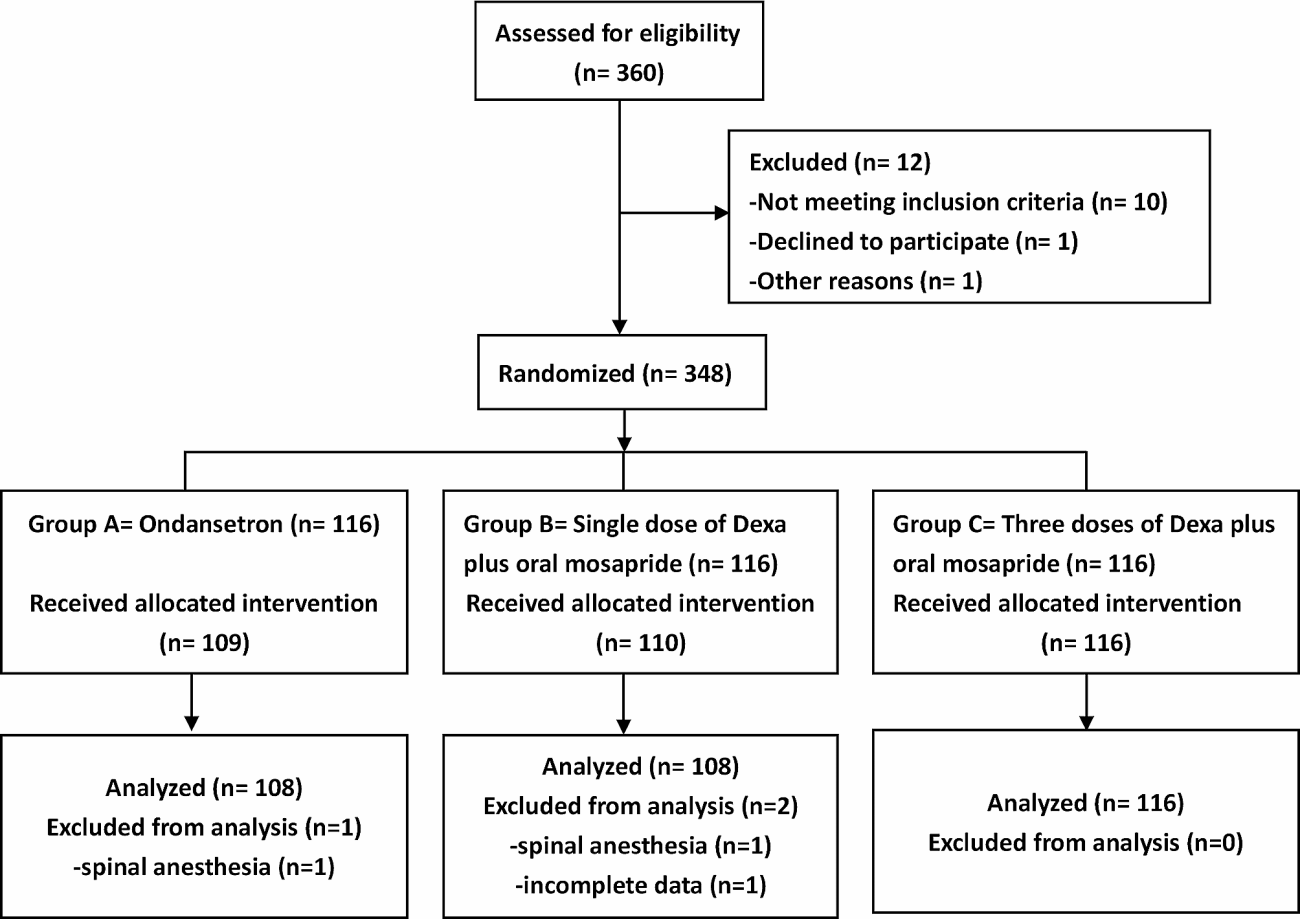
A flow diagram shows the patients recruitment

In total, 348 patients were initially allocated to Groups A, B, and C (116 patients in each group). We excluded eight patients each from Groups A and B according to the defined exclusion criteria. Thus, 332 patients (108 in Group A, 108 in Group B, and 116 in Group C) were included in the final analysis (Fig. 1). We found no differences in demographic characteristics or clinical data among the groups (Table 1).

**Table 1:** Baseline characteristics of all patients

	Group A (*n* = 108)	Group B (*n* = 108)	Group C (*n* = 116)	p value
Age (years)	57.4 ± 14.2	62.2 ± 12.7	61.2 ± 13.3	0.087
BMI (kg/m^2^)	23.9 ± 4.1	24.1 ± 3.2	24.1 ± 3.9	0.899
Female / Male	74 / 34	75 / 33	80 / 36	0.993
Smoking (n, %)	34 (31.5%)	35 (32.4%)	22 (18.2%)	0.164
History of motion sickness				0.506
YES	44	42	36	
NO	64	66	80	
ASA Score				0.813
ASA = 2	76	75	86	
ASA = 3	32	33	30	
Type of surgery				
THA	52	45	64	0.245
TKA	56	63	52	
Number of comorbidities				0.518
1	42	52	50	
≥ 2	8	12	14	
Operation time (min)	77.4 ± 20.9	77.7 ± 26.2	78.3 ± 19.3	0.973
Anesthesia time (min)	126.7 ± 28.7	123.9 ± 31.1	122.1 ± 22.0	0.688
PACU time (min)	72.7 ± 47.4	78.0 ± 38.9	75.0 ± 36.0	0.72
Sufentanil (ug)	24.3 ± 3.4	24.6 ± 3.3	24.6 ± 5.6	0.888
Remifentanil (mg)	0.6 ± 0.1	0.6 ± 0.2	0.6 ± 0.3	0.530
Propofol (mg)	202.8 ± 109.4	206.0 ± 129.9	225.0 ± 122.1	0.565
Midazolam (mg)	2.1 ± 0.5	2.1 ± 0.5	2.3 ± 0.8	0.059
Atracurium (mg)	13.6 ± 2.6	13.9 ± 3.0	13.3 ± 3.8	0.511
Sevoflurane (ml)	30.8 ± 6.6	30.2 ± 9.5	28.6 ± 7.2	0.341

ASA, American Society of Anesthesiologists; BMI, body mass index; THA, total hip arthroplasty; TKA, total knee arthroplasty; PACU, post-anesthesia care unit

During the whole evaluation period, the patients in Group C experienced lowest incidence of total PONV (29.3%), nausea (15.5%) and vomiting (13.8%, Table 2). Similarly, more patients who received three doses of dexamethasone and mosapride in Group C achieved a complete response (70.7%) compared with Groups A (48.2%, *p* = 0.001) and B (63.0%, *p* = 0.219). In addition, fewer patients in Group C had severe PONV (4.3%) compared with Group B (20.4%, *p* < 0.001) and Group A (25.9%, *p* < 0.001).

**Table 2:** Incidence of PONV during the first postoperative 48 h

	Group A (*n* = 108)	Group B (*n* = 108)	Group C (*n* = 116)	P*	p1^†^	p2^†^	p3^†^
Total PONV	56(51.9%)	40(37.0%)	34(29.3%)	0.002	0.028	**0.001**	0.219
Vomiting	22 (20.4%)	17 (15.7%)	16 (13.8%)	0.401	-	**-**	-
Nausea	34 (31.5%)	23 (21.3%)	18 (15.5%)	0.016	0.086	**0.005**	0.264
Complete response	52 (48.2%)	68 (63.0%)	82 (70.7%)	0.002	0.028	**0.001**	0.219
Mild PONV	10 (9.3%)	6 (5.6%)	16 (13.8%)	0.112	-	**-**	-
Moderate PONV	18 (16.7%)	12 (11.1%)	13 (11.2%)	0.375	-	**-**	-
Severe PONV	28 (25.9%)	22 (20.4%)	5 (4.3%)	< 0.001	0.333	**< 0.001**	**< 0.001**
Moderate + severe PONV	46(42.6%)	34 (31.5%)	18 (15.5%)	< 0.001	0.082	**< 0.001**	**0.005**

Data are presented as number of patients (percentage)

PONV, postoperative nausea and vomiting

*Uncorrected p values (for the three-way comparison)

^**†**^Bonferroni-corrected p values: p1, Group A vs. B; p2, Group A vs. C; p3, Group B vs. C. The corrected significance threshold was 0.016

According to the timing and duration of PONV, concurrent use of three doses of dexamethasone, mosapride and ondansetron mainly reduced the incidence of PONV from postoperative 6 to 48 h (Table 3). Of all patients with episodes of PONV in Group C, 88% of patients had episodes that lasted < 6 h, which was a higher proportion than in Group B (35%, *p* = 0.005) and Group A (21%, *p* = 0.015, Table 4). And the patients had episodes that lasted ≥ 12 h was less in Group C than group A and B (0% vs. 50% vs. 42.5%, *p*<0.05 for all). The mean time of PONV in Group C (2. 9 ± 1.7 h) was shorter than that in Groups A (16.2 ± 5.0 h, *p* = 0.002) and B (18.2 ± 8.2 h, *p* = 0.001).

**Table 3:** Timing of PONV events during the first postoperative 48 h

Time	Total (*n* = 332)	Group A (*n* = 108)	Group B (*n* = 108)	Group C (*n* = 116)	p*	p1^†^	p2^†^	p3^†^
0–6 h	75 (22.6%)	19 (17.6%)	27 (25%)	29 (25%)	0.319	**-**	**-**	-
6–12 h	28 (8.4%)	15 (13.9%)	8 (7.4%)	5(4.3%)	0.032	0.123	**0.012**	0.322
12–24 h	17 (5.1%)	13 (12.0%)	4 (3.7%)	0	< 0.001	0.023	**0.001**	0.052
24–48 h	16 (4.8%)	13 (12.0%)	3 (2.8%)	0	< 0.001	**0.009**	**< 0.001**	0.110

Data are presented as number of patients (percentage)

PONV, postoperative nausea and vomiting

*Uncorrected p values (for the three-way comparison)

^**†**^Bonferroni-corrected p values: p1, Group A vs. B; p2, Group A vs. C; p3, Group B vs. C. The corrected significance threshold was 0.016

**Table 4:** Duration of PONV during the first postoperative 48 h

Duration	Total (*n* = 332)	Group A (*n* = 108)	Group B (*n* = 108)	Group C (*n* = 116)	p*	p1^†^	p2^†^	p3^†^
Mean time (h)	13.6 ± 5.4	16.2 ± 5.0	18.2 ± 8.2	2. 9 ± 1.7	0.002	0.606	**0.002**	**0.001**
< 6 h	56 (16.9%)	12 (11.1%)	14 (13.0%)	30 (25.9%)	0.005	0.676	**0.005**	**0.015**
6–12 h	29 (8.7%)	16 (14.8%)	9 (8.3%)	4 (3.4%)	0.010	0.137	0.003	0.118
12–24 h	18 (5.4%)	11(10.1%)	7 (6.5%)	0	0.003	0.325	**< 0.001**	**0.005**
> 24 h	27 (8.1%)	17 (15.7%)	10 (9.3%)	0	< 0.001	0.150	**< 0.001**	**0.002**

Data are presented as number of patients (percentage) or mean ± standard deviation

PONV, postoperative nausea and vomiting

*Uncorrected p values (for the three-way comparison)

^**†**^Bonferroni-corrected p values: p1, Group A vs. B; p2, Group A vs. C; p3, Group B vs. C. The corrected significance threshold was 0.016

With the use of multiple doses of dexamethasone, fewer patients needed antiemetic rescue treatment in Group C. The total consumption of Metoclopramide in Group C (1.4 ± 0.5 mg) was less than that in Group A (6.5 ± 1.1 mg, *p* = 0.017) and B (2.9 ± 1.2 mg, *p* = 0.415), however, the total consumption of Ondansetron was not significantly different. Moreover, the consumption of oxycodone and pethidine in Group C (1.8 ± 0.3 mg, 6.0 ± 1.0 mg) was less than that in Groups B (17.9 ± 9.7 mg, *p* < 0.001; 20.6 ± 9.8 mg, *p* = 0.005, respectively) and A (33.2 ± 8.7 mg, *p* < 0.001; 20.4 ± 8.3 mg, *p* = 0.001; respectively, Table 5).

**Table 5:** Requirement of rescue treatment for PONV and pain during the postoperative 48 h

	Group A (*n* = 108)	Group B (*n* = 108)	Group C (*n* = 116)	p*	p1^†^	p2^†^	p3^†^
Metoclopramide (mg)	6.5 ± 1.1	2.9 ± 1.2	1.4 ± 0.5	0.047	0.055	**0.017**	0.415
Ondansetron (mg)	1.7 ± 0.5	1.7 ± 0.5	0.6 ± 0.1	0.238	-	-	-
Oxycodone (mg)	33.2 ± 8.7	17.9 ± 9.7	1.8 ± 0.3	< 0.001	0.001	**< 0.001**	**< 0.001**
Pethidine (mg)	20.4 ± 8.3	20.6 ± 9.8	6.0 ± 1.0	0.002	0.959	**0.005**	**0.001**

Data are presented as mean ± standard deviation

PONV, postoperative nausea and vomiting

*p values with one-way analysis of variance; ^**†**^p values with Tukey’s post hoc test

P1, Group A vs. B; p2, Group A vs. C; p3, Group B vs. C

The time until first defecation was shortest in Group C than group A and B (37.2 ± 9.0 h, vs. 52.4 ± 16.5 h, vs. 46. 7 ± 11.8 h) and all intergroup differences were statistically significant (*p* < 0.05 for all) (Table 6). Moreover, the patients in Group C had a better appetite on POD 1 (2.7 ± 0.5, vs. 2.4 ± 0.7, vs. 2.3 ± 0.7), were more satisfied with their healthcare (9.7 ± 0.4, vs. 9.3 ± 0.9, vs. 9.3 ± 0.7), and had a shorter length of hospitalization (4.1 ± 2.2 d, vs. 5.0 ± 2.2 d, vs. 5.4 ± 2.1 d) than the patients in Groups B and A (*p* < 0.05 for all).

**Table 6:** Other clinical outcomes

	Group A (*n* = 108)	Group B (*n* = 108)	Group C (*n* = 116)	p	p1	p2	p3
Time to first defecation (h)	52.4 ± 16.5	46. 7 ± 11.8	37.2 ± 9.0	< 0.001	**0.006**	**< 0.001**	**< 0.001**
Appetite score on POD 0	2.2 ± 0.8	2.4 ± 0.7	2.6 ± 0.6	0.009	0.106	0.002	0.054
Appetite score on POD 1	2.3 ± 0.7	2.4 ± 0.7	2.7 ± 0.5	0.001	0.298	**< 0.001**	**0.002**
Appetite score on POD 2	2.6 ± 0.5	2.6 ± 0.5	2.7 ± 0.4	0.121	-	-	-
Time to first ambulation (h)	27.1 ± 8.2	28.5 ± 10.8	25.6 ± 6.3	0.298	-	-	-
Satisfaction score	9.3 ± 0.7	9.3 ± 0.9	9.7 ± 0.4	0.001	0.661	**0.007**	**< 0.001**
LOH (d)	5.4 ± 2.1	5.0 ± 2.2	4.1 ± 2.2	0.02	0.321	**0.001**	**0.049**

Data are presented as mean ± standard deviation

POD, postoperative day; LOH, length of hospitalization

*p values with one-way analysis of variance; ^**†**^p values with Tukey’s post hoc test

P1, Group A vs. B; p2, Group A vs. C; p3, Group B vs. C

Among patients with diabetes mellitus, the fasting blood glucose level was higher in Group C than in Group A on POD 1 (11.0 ± 2.9 vs. 9.5 ± 2.2 mmol/L, *p* = 0.001; Table 7). Among patients without diabetes mellitus, the fasting blood glucose level was higher in Group C than Groups A and B on POD 1 (7.8 ± 1.3 vs. 6.8 ± 1.2, *p* = 0.017; vs. 6.9 ± 1.6 mmol/L, *p* = 0.041; respectively) and POD 2 (7.1 ± 1.2 vs. 5.6 ± 1.0 mmol/L, *p* = 0.039; vs. 5.7 ± 1.8 mmol/L, *p* = 0.048; respectively), although the blood glucose level was acceptable in all groups. During the study and follow-up period, the incidence of complications was comparable among the three groups.

**Table 7:** Incidence of complications and level of blood glucose on POD 1 and 2

	Group A (*n* = 108)	Group B (*n* = 108)	Group C (*n* = 116)	p*	p1^†^	p2^†^	p3^†^
Diabetes Mellitus patients (mmol/ L)							
FBG on POD1	9.5 ± 2.2	10.7 ± 2.4	11.0 ± 2.9	0.001	0.001	**0.001**	0.525
2-h PBG after breakfast	11.6 ± 1.5	12.3 ± 3.1	14.9 ± 3.9	0.002	0.177	**0.001**	.**001**
2-h PBG after lunch	11.5 ± 1.3	13.2 ± 3.7	14.8 ± 2.6	0.096	-	-	-
2-h PBG after dinner	11.0 ± 2.3	12.7 ± 3.2	10.1 ± 2.7	0.173	-	-	-
FBG on POD2	9.4 ± 1.0	9.8 ± 2.1	7.7 ± 3.1	0.127	-	-	-
Non- Diabetes Mellitus patients (mmol/ L)							
FBG on POD1	6.8 ± 1.2	6.9 ± 1.6	7.8 ± 1.3	0.021	0.212	**0.017**	**0.041**
FBG on POD2	5.6 ± 1.0	5.7 ± 1.8	7.1 ± 1.2	0.031	0.76	**0.039**	**0.048**
Prolonged QT syndrome	0	0	0	-			
Wound site discharge	2 (1.9%)	3 (2.8%)	4 (3.5%)	0.913	-	-	-
Surgical site infection	0	0	0	-			
Pulmonary infection	2 (1.9%)	6 (5. 6%)	1 (0.9%)	0.104	-	-	-
Re-admission	1 (0.9%)	1 (0.9%)	1 (0.9%)	1.000	-	-	-

Data are presented as mean ± standard deviation or number (percentage)

POD, postoperative day; FBG, fasting blood glucose; PBG, 2-hour postprandial blood glucose

*p values with one-way analysis of variance; ^**†**^p values with Tukey’s post hoc test

p1, Group A vs. B; p2, Group A vs. C; p3, Group B vs. C

## Discussion

As an important part of an enhanced recovery after surgery program, PONV management remains a challenge during the postoperative period, especially in the setting of general anesthesia. Therefore, we should pay more attention to PONV prevention and treatment because PONV can cause anxiety and loss of appetite and can increase the risk of complications and prolong length of hospital stay. Although some clinical guidelines and suggestions have been recommended, the incidence of PONV remains a “little big” problem because of the lack of an ideal PONV prophylaxis regimen.

In the present study, we compared three different PONV prophylactic protocols and found that with the use of ondansetron, oral mosapride and three doses of dexamethasone, patients experienced a lower incidence of nausea and severe PONV, a shorter PONV duration time, a better appetite, and a higher satisfaction score. More importantly, the combined use of three doses of dexamethasone, ondansetron, and mosapride reduced the consumption of opioids and promoted postoperative bowel function recovery, thus reducing the incidence of postoperative constipation.

Effective treatments that limit PONV allow patients to mobilize earlier, decrease the length of stay, and improve patients’ overall satisfaction. Many factors are attributable to the incidence of PONV, including female sex, a history of PONV or motion sickness, and opioid use [16, 17]. 5-HT_3_ receptor antagonists and glucocorticoids are the most common treatment options for PONV prevention. Ondansetron, one type of 5-HT_3_ receptor antagonist, has been recommended as the first-line agent for PONV prevention by some clinical guidelines [4]. Additionally, a study by Apfel et al. [18] showed comparable efficacy in reducing the incidence of PONV between 4 mg ondansetron and 4 mg dexamethasone. For this reason, our control group comprised patients treated with ondansetron only.

Dexamethasone is a high-potency, long-acting glucocorticoid with good bioavailability and few corticoid-related adverse effects [19]. Glucocorticoids reduce PONV through a central antiemetic effect by inhibiting prostaglandin synthesis and release of endogenous opioids. Another reason for the antiemetic effect of dexamethasone is the anti-inflammatory response that inhibits the release and reduces the levels of inflammatory factors such as C-reactive protein and interleukin-6 [7–9]. Our research team systematically explored the potential clinical benefit of multiple low doses of dexamethasone, including two or three doses in the setting of total hip and knee arthroplasty [7–9, 20]. In studies performed by Xu et al. [7] and Lei et al. [8], two doses of 10 mg dexamethasone were intravenously administered upon anesthetic induction and return to the inpatient unit. Two other studies by the same research teams evaluated the efficacy and safety of three doses of dexamethasone (10 or 20 mg at anesthetic induction, 10 mg after returning to the inpatient unit, and 10 mg at 24 h after the first dose) following primary total hip and knee arthroplasty [9, 20]. In all of the aforementioned studies, the authors focused on the effect of dexamethasone on the postoperative inflammatory response, pain relief, and joint function. All studies also indicated promising effects of three doses of dexamethasone. In contrast to these previous studies, the current study mainly focused on the outcomes of PONV, appetite, bowel function, and safety. Moreover, the prophylactic regimen in the current study was different from that in the previous studies; i.e., the current regimen combined dexamethasone with a 5-HT_4_ agonist at different time intervals. Additionally, we compared the new regimen with other common regimens (ondansetron only and a single dose of dexamethasone). This is a major strength of our study.

Another advantage of dexamethasone for PONV prevention is the additional anti-inflammatory effect. A previous study demonstrated that PONV is specific to the patients’ perioperative inflammatory control [21], which would peak at postoperative 24–48 h, and last for at least 72 h. Systematic reviews and meta-analyses have also shown that a high dose of dexamethasone (>10 mg or >0.1 mg/kg) was more effectively to reduce the incidence of PONV and provide better postoperative pain control [22, 23]. In addition, the inflammation was closely related with fibrinolysis, which would peak at postoperative 6 h and last for 24 h. And similar to another study [24], our preliminary results indicated that most of the PONV episodes (87%) occurred during the first 12 h postoperatively. Therefore, we hypothesized that repeated dose at postoperative 6 and 24 h would be more effective although the half- life of dexamethasone is 36 h. Our results also revealed a lower incidence of severe PONV (4.3%) and a shorter duration (2.9 h) in the patients treated with three doses of dexamethasone. Moreover, the patients in this group (Group C) experienced better pain control with respect to less opioid consumption (Table 5), and this also contributed to the lower incidence of PONV. However, we can’t conclude whether the lower incidence of PONV was owed to the first or second or third dose of dexamethasone.

Mosapride, a 5-HT_4_ receptor agonist, is a gastroprokinetic agent indicated for the treatment of gastrointestinal symptoms such as heartburn, nausea/vomiting associated with chronic gastritis, and functional dyspepsia. The in vivo study by Mine et al. [25] showed that mosapride was helpful to improve the selective serotonin reuptake inhibitor (SSRI)-induced delay in gastric emptying and the incidence of SSRI-induced emesis, which was in accordance with another clinical study [26]. Moreover, another study also showed the potential anti-emetic effect of mosapride in patients undergoing chemotherapy [13]. Interestingly, in the present study, the patients in Group C (oral mosapride in addition to three doses of dexamethasone) had a shorter duration of time passed until the first defecation. This raises the possibility that oral mosapride is helpful to reduce the incidence of PONV by promoting gastrointestinal motility. Although our results indicated no adverse events such as prolonged QT syndrome associated with mosapride, in view of the fact that another 5-HT_4_ receptor agonist, cisapride, was withdrawn because of severe cardiotoxicity, further studies are warranted to investigate the potential antiemetic efficacy and safety profile of mosapride in the setting of joint arthroplasty before routine recommendation.

In addition to the fewer episodes and decreased severity of PONV in Group C, these patients had better bowel function, higher appetite scores, shorter hospital stays, and higher satisfaction scores. This might have been partially due to the application of mosapride, although multiple doses of dexamethasone could have improved the pain control, joint function, early ambulation, and length of hospital stay.

The main obstacle to the widespread application of dexamethasone is the concern about its safety, especially in terms of the risk of infection and hyperglycemia. Although the literature has provided some evidence that the use of low-dose dexamethasone does not increase the risk of adverse events [27, 28], the current results were inconclusive. In our study, the fasting blood glucose level in patients both with and without diabetes receiving dexamethasone significantly increased, but it remained at an acceptable level. Additionally, the incidence of wound complications during the follow-up period was comparable among the three groups. Nevertheless, we cannot conclude that the use of dexamethasone is safe because our study sample was not large enough to detect statistical significance. A previous power analysis showed that > 3500 patients would be required to meaningfully evaluate an increase in surgical infection [22]. Thus, we must acknowledge that this study lacks the power to fully assess the low incidence of events because of the relatively small sample size. The rate of such negative outcomes could be much more severe than what we expected, and the results should be interpreted with caution. Although large-scale prospective studies are still needed, our study might provide some insight into the safety of dexamethasone.

We acknowledge that our study has some limitations. First and foremost, the generalizability of this regimen was limited because of same postoperative pain management and anesthesia protocol. Different from most western countries, general anesthesia was preferable by the anesthesia team in our institution because of better feasibility of early ambulation and anticoagulation. Second, we did not evaluate the effect of this regimen on the postoperative inflammatory response and other functional outcomes because previous studies have confirmed its efficacy in this regard [7–9]. Thirdly, the follow-up period was too short to detect the possible infectious complications associated with dexamethasone. The last but not the least, the interventions between the three groups were not definitely comparable and consistent, especially the lack of isolation of mosapride, which this made us difficult to conclude the prophylactic role of mosapride in the prevention of PONV. Furthermore, because of confounding bias, we couldn’t conclude how much PONV prevention effect was related to mosapride or dexamethasone. On the other hand, as mentioned above, because of the lack of direct evidence of mosapride on PONV and the effect of dexamethasone on consumption of opioid agents, we also can’t make the exact conclusion whether the decrease of PONV was a primary effect of the drug regimen or the secondary outcome of less opioid consumption. Nonetheless, in our opinion, the observed clinical outcomes result from the direct and indirect role of this combined regimen.

## Conclusion

Combined use of ondansetron, mosapride and three doses of dexamethasone effectively reduced the incidence of PONV. Additionally, this regimen resulted in a better postoperative appetite, bowel function recovery, and pain relief when compared with a single dose or ondansetron only in patients undergoing primary total joint arthroplasty under general anesthesia.

### Electronic supplementary material

Below is the link to the electronic supplementary material.

Supplementary Material 1

## Data Availability

The datasets used and analyzed during the current study are available from the corresponding author on reasonable request.

## Abbreviations

PONV: postoperative nausea and vomiting
THA: total hip arthroplasty
TKA: total knee arthroplasty
ASA: American Society of Anesthesiologists
PACU: post-anesthesia care unit
BMI: body mass index
